# Neuroprotective effects of apigenin on retinal ganglion cells in ischemia/reperfusion: modulating mitochondrial dynamics in in vivo and in vitro models

**DOI:** 10.1186/s12967-024-05260-1

**Published:** 2024-05-13

**Authors:** Jiawen Wu, Daowei zhang, Hongli Liu, Jufeng Li, Ting Li, Jihong Wu, Shenghai Zhang

**Affiliations:** 1 Eye Institute, Eye and ENT Hospital, College of Medicine, Fudan University, Shanghai, China; 2 Shanghai Key Laboratory of Visual Impairment and Restoration, Science and Technology Commission of Shanghai Municipality, Shanghai, China; 3 State Key Laboratory of Medical Neurobiology, Institutes of Brain Science and Collaborative Innovation Center for Brain Science, Shanghai, China; 4 Key Laboratory of Myopia, Ministry of Health, Shanghai, China

**Keywords:** Apigenin, Retina ischemia/reperfusion, Retinal ganglion cells, Mitochondrial dynamics, Neuroprotection

## Abstract

**Background:**

Retinal ischemia/reperfusion (RIR) is implicated in various forms of optic neuropathies, yet effective treatments are lacking. RIR leads to the death of retinal ganglion cells (RGCs) and subsequent vision loss, posing detrimental effects on both physical and mental health. Apigenin (API), derived from a wide range of sources, has been reported to exert protective effects against ischemia/reperfusion injuries in various organs, such as the brain, kidney, myocardium, and liver. In this study, we investigated the protective effect of API and its underlying mechanisms on RGC degeneration induced by retinal ischemia/reperfusion (RIR).

**Methods:**

An in vivo model was induced by anterior chamber perfusion following intravitreal injection of API one day prior to the procedure. Meanwhile, an in vitro model was established through 1% oxygen and glucose deprivation. The neuroprotective effects of API were evaluated using H&E staining, spectral-domain optical coherence tomography (SD-OCT), Fluoro-Gold retrograde labeling, and Photopic negative response (PhNR). Furthermore, transmission electron microscopy (TEM) was employed to observe mitochondrial crista morphology and integrity. To elucidate the underlying mechanisms of API, the terminal deoxynucleotidyl transferase dUTP nick end labeling (TUNEL) assay, flow cytometry assay, western blot, cell counting kit-8 (CCK-8) assay, lactate dehydrogenase (LDH) assay, JC-1 kit assay, dichlorofluorescein-diacetate (DCFH-DA) assay, as well as TMRE and Mito-tracker staining were conducted.

**Results:**

API treatment protected retinal inner plexiform layer (IPL) and ganglion cell complex (GCC), and improved the function of retinal ganglion cells (RGCs). Additionally, API reduced RGC apoptosis and decreased lactate dehydrogenase (LDH) release by upregulating Bcl-2 and Bcl-xL expression, while downregulating Bax and cleaved caspase-3 expression. Furthermore, API increased mitochondrial membrane potential (MMP) and decreased extracellular reactive oxygen species (ROS) production. These effects were achieved by enhancing mitochondrial function, restoring mitochondrial cristae morphology and integrity, and regulating the expression of OPA1, MFN2, and DRP1, thereby regulating mitochondrial dynamics involving fusion and fission.

**Conclusion:**

API protects RGCs against RIR injury by modulating mitochondrial dynamics, promoting mitochondrial fusion and fission.

**Supplementary Information:**

The online version contains supplementary material available at 10.1186/s12967-024-05260-1.

## Introduction

Retinal ischemia/reperfusion (RIR) is involved in a series of ocular diseases, such as glaucoma, hypertensive retinopathy, diabetic retinopathy (DR), and retinal central/branch arterial/vein occlusion [1–5]. RIR injury triggers a cascade of events that induces cell death, culminating in retinal ganglion cell (RGC) death and vision loss [1]. However, current clinical treatments lack practical neuroprotective agents specifically targeting the survival of actual RGCs or bipolar cells, as many of which are experimental [6, 7]. Therefore, therapeutic approaches aiming to improve the resistance of RGCs to RIR are needed.

Mitochondrial dynamics maintain normal mitochondrial function, which requires the participation of mitochondrial fusion and fission [8], and are involved in a variety of cellular processes, such as the cell cycle, apoptosis, cell migration, mitophagy, and reactive oxygen species (ROS) production [9]. Fusion is triggered by several GTPases, such as mitofusin (MFN) in the outer membrane and optic atrophy 1 (OPA1) in the inner membrane, whereas fission is triggered by dynamin-related protein-1 (DRP1) in the outer membrane [10, 11]. Excessive or untimely fission or fusion is detrimental to mitochondrial quality and homeostasis [12]. Recent research has reported that RGCs are sensitive to alterations in mitochondrial dynamics and function [10, 11]. A fine balance between mitochondrial fusion and fission is essential for the survival and optimal function of RGCs.

Apigenin (API, 4′,5,7-trihydroxyflavone), a member of the flavonoid subgroup flavones, can be found in vegetables, fruits, herbs, and plant-based beverages [13, 14]. It has been studied in neurodegenerative diseases, such as cerebral ischemic injury, Alzheimer’s disease, and depression [13, 15]. API has a better safety profile than other flavonoids, such as quercetin and kaempferol [16–18]. The recently reported protective effect of API against light-induced damage to the retina and Nrf KO mouse retina provides a reference for the use of API in fundus diseases [19, 20]. However, to the best of our knowledge, no reports have demonstrated the effects of API on RIR-induced RGC degeneration.

In this study, we studied the potential of API as a treatment for RIR-induced RGC damage by investigating its effects on retinal morphology, RGC survival, and mitochondrial integrity and function.

## Materials and methods

### Retinal ischemic/reperfusion

Animal experiments were performed in accordance with the Guide for the Care and Use of Laboratory Animals published by the US National Institutes of Health (NIH Publication No. 85 − 23, revised 2011) and the guidelines on the ethical use of animals of Fudan University. Adult male Wistar rats (180–200 g; SLAC Laboratory Animal Co., Ltd., Shanghai, China) were housed under a daily 12 h light/12 h dark cycle with free access to food and water. For the induction of retinal ischemia, as previously described [21], the rats were deeply anesthetized with 1% sodium pentobarbital for 30 min. Intraocular pressure (IOP) was increased to 75 ± 5 mmHg (TonoLab, Icare, Vantaa, Finland) for 1 h by anterior chamber perfusion using a 30-gauge needle in the anterior chamber without injuring the lens. Experiments were performed under normal lighting conditions in the early afternoon. The rats’ eyes were treated with tropicamide to dilate the pupils, lidocaine hydrochloride to provide local anesthesia, tobramycin to prevent infection, and carbomer to prevent cornea edema. A sham operation was performed on the contralateral eye without elevating IOP. IOP was normalized after 60 min. Then, tobramycin ointment was used to prevent bacterial infection.

### In vivo experimental group categorization

**Fig. 1:**
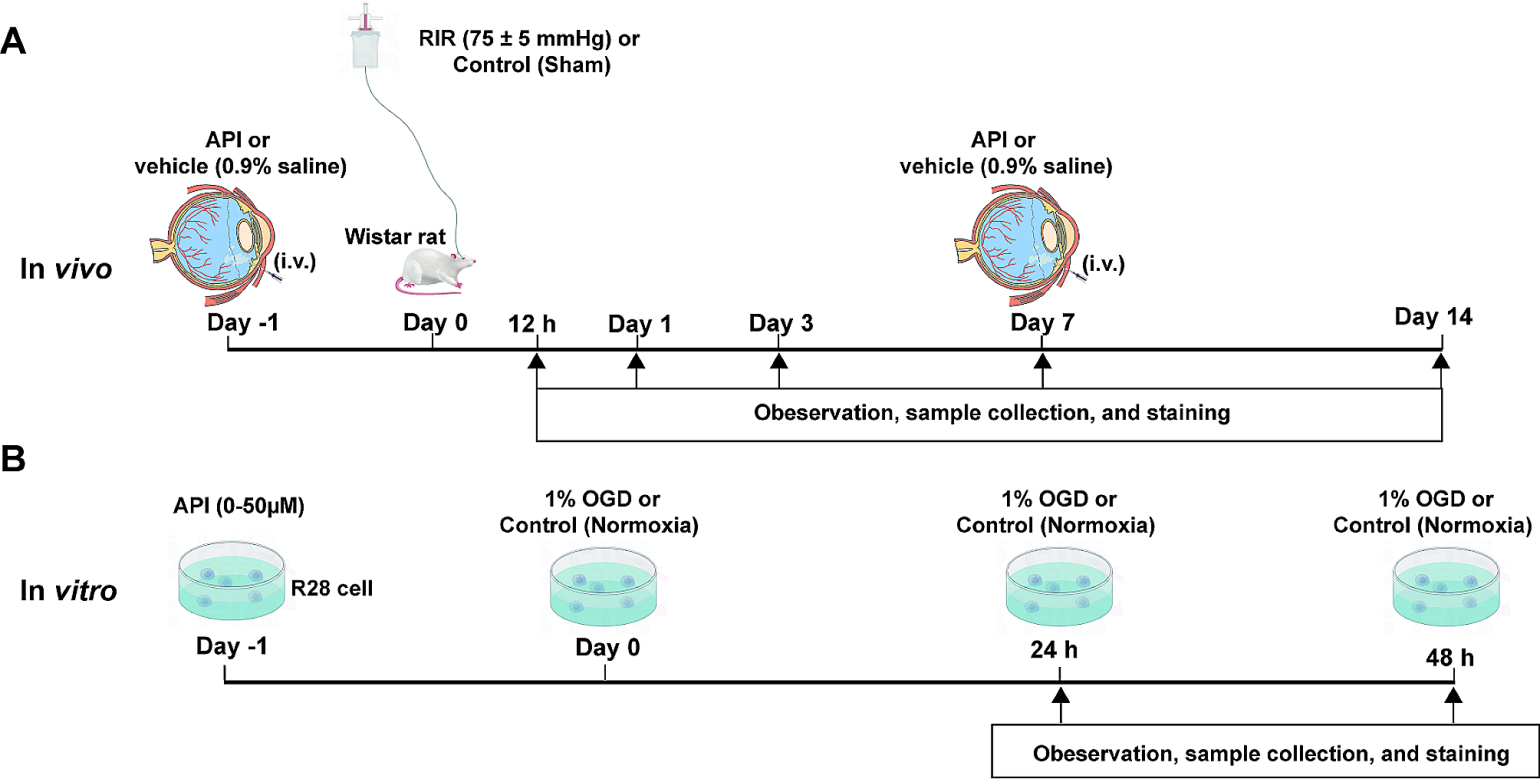
Diagram of the groups and time points In Vivo and In Vitro. API, apigenin; i.v., intravitreal injection; OGD, oxygen-glucose deprivation

The rats were adaptively fed for 7 days and randomly divided into several groups at different times: control (*n* = 35), control + API (*n* = 4), RIR (*n* = 27), vehicle (*n* = 38), and API (100µM, 500µM, 1mM) groups (*n* = 50). The control group represented Wistar rats that underwent a sham operation on the eye. The control + API group represented rats subjected to intravitreal injection of API after a sham operation. The RIR group consisted of rats subjected to RIR operation. The vehicle group indicated rats that received intravitreal injections of saline (0.9%) solution before undergoing RIR treatment. The API (100µM, 500µM, 1mM) groups were subjected to RIR treatment after intravitreal injection of the corresponding concentration of API. Depending on the experimental requirements, the number of experimental animals varied, ranging from 3 to 6 (supplementary file 1). The seventh day after RIR surgery was the time point for detecting effective indicators, while the 14th day was for long-term observation [22, 23]. Data were collected from only one eye of each rat. A detailed diagram was shown in Fig. 1.

### Intravitreal injection

API (Item No. HY-N1201) with purity higher than 99% was purchased from MCE, dissolved in DMSO to a stock concentration of 100 mM, and stored at − 20 °C for use within 1 month after preparation. When used, the API stock solution was diluted to the corresponding concentration, and the proportion of DMSO was 1%. A total of 2 µl of 100 µM–1 mM (0.2–2 nmol/eye) API was intravitreally injected 1 day in advance. In detail, the rats’ eyes were treated with tropicamide to dilate the pupils and with lidocaine hydrochloride to provide local anesthesia. API was administered to the vitreous 2–3 mm behind the corneal limbus. Damage to the lens and retina was avoided. The needle was retained for 10 s after injection to prevent the overflow of API. After the injection, the rats with lens and retinal injuries were removed. They were anesthetized with 1% sodium pentobarbital, then their eyeballs were processed with excessive chloral hydrate to death for further hematoxylin and eosin (H&E) staining, TdT-mediated dUTP nick-end labeling (TUNEL), and Western blot analysis at an appropriate time.

### RGC labeling and survival quantitation

The rats were deeply anesthetized with 1% sodium pentobarbital and placed in a stereotaxic instrument. Hydroxytoluene indapamide (3%, fluoro-gold [FG], Sigma-Aldrich) was dissolved in 90% PBS and 10% DMSO. As previously described [24], double-sided holes were drilled on the surface of the superior colliculus. Approximately 2 µl of FG solution was injected into each superior colliculus at the following locations: 6.0 mm posterior to the bregma and 1.2 mm lateral to the midline. After injection, the microsyringe was retained for 5 min and slowly removed. Finally, tobramycin ointment was applied topically, and the dissected scalp was sutured. FG was then absorbed by RGC axons and transported retrogradely to the retinal soma. After 7 days, the animal was euthanized, and its whole retina was carefully dissected and laid flat. Off-center and peripheral images were obtained from eight quadrants (upper, lower, nose, and temporal). The average number of RGCs of the off-central and peripheral retina were counted manually by using ImageJ software (NIH, Bethesda, MD, United States).

### Spectral-domain optical coherence tomography and fundus photography

The rats were intraperitoneally anesthetized with 1% sodium pentobarbital, and their pupils were dilated by using topical 1.0% tropicamide. Carbomer ophthalmic gel (Bausch & Lomb) was applied to the cornea surface. Fundus photography was performed by using a retinal imaging system (Optoprobe, Pontypridd, Mid Glamorgan, UK). A spectral-domain optical coherence tomography retinal imaging system (OPTO-RIS, OPTOPROBE, Optoprobe Science LTD, UK) was then applied for focusing and image acquisition. The instrument’s built-in software (Version 2.0, Optoprobe, Pontypridd, Mid Glamorgan, UK) automatically estimated the thickness of each layer of the retina at the same distance from the optic nerve head. The deviation value was manually corrected.

### H&E staining

Eyes were enucleated at the appropriate time points after RIR and immediately placed in 4% PFA for 24 h. Then, the eyes were embedded in paraffin, sectioned to a thickness of 4 μm, and stained with H&E. Sections were examined by blinded observers by using a light microscope (Leica, Wetzlar, Germany), and cell counts were quantitated. Specifically, the number of cells in the ganglion cell layer of each group was counted at the same distance from the optic nerve head in a region spanning 300 μm and manually measured per area by using Image J as described in a previous article [25].

### Electroretinogram recordings

The photopic negative response (PhNR) procedure was conducted by using an Espion Diagnosys system (Diagnosys, Littleton, MA, USA) as previously described [26]. Briefly, the rats were intraperitoneally injected with 1% sodium pentobarbital. Their pupils were dilated with tropicamide (0.5%). Then, the PhNR procedure was performed. Data were collected and analyzed by using GraphPad Prism 9.

### TUNEL assay

After retinal paraffin sections were repaired through dewaxing, cell apoptosis was detected by performing TUNEL assays with an In Situ Cell Death Detection Kit (TMR Red, Roche, Mannheim, Germany) in accordance with the manufacturer’s instructions. Sections were counterstained with DAPI and observed by using a Panoramic 250 Flash II Slide Scanner (3DHISTECH, Budapest, Hungary), and images were collected.

### Apoptosis flow cytometry assay

Apoptosis was detected by using Annexin V–allophycocyanin (APC)/7-aminoactinomycin D (AAD) (BD Biosciences). In detail, retinal tissue was washed three times and cut into small pieces 2–4 mm in size. The tissue was then digested into single cells in trypsin–EDTA solution (Gibco, Thermo Fisher Scientific, Waltham, MA, United States) containing 0.25% trypsin and 0.02% EDTA at 37 °C for 20 min. The cells were harvested and added with APC and AAD in accordance with the manufacturer’s instructions. Cell samples were analyzed via flow cytometry (FACScan, BD Biosciences), and apoptotic fractions were recorded.

### Transmission electron microscopy

As previously described [25], the retina was quickly removed for fixation and sequentially dehydrated, embedded, sectioned, and observed under a transmission microscope (TEM; HITACHI, HT7700). Mitochondrial crista morphology and mitochondrial integrity were determined in accordance with previous reports and analyzed by using ImageJ software [27].

### 1% Oxygen-glucose deprivation model

The R28 retinal cell line, an adherent retinal precursor cell line obtained from day 6 Sprague-Dawley rat retina, has been extensively characterized and utilized in both in vitro and in vivo studies of retinal cell behavior, neuroprotection, and cytotoxicity, and possesses the characteristics of both RGC and glial cells [28]. R28 cells were cultured in a T25 flask with low glucose DMEM (Hyclone), 10% fetal bovine serum, 100 U/mL penicillin, and 100 mg/mL streptomycin and grown in 95% air and 5% CO_2_ humidified atmosphere at 37 ℃. The 1% oxygen-glucose deprivation **(**1% OGD) model was first cultivated in a standard culture environment to a density of approximately 60–70%, then transferred to a sugar-free medium (Gibco) and a hypoxic incubator with 1% oxygen concentration [29].

### Cell viability

Approximately 1 × 10^5^ R28 cells were cultured in 96-well plates to 60–70% confluence under normal conditions. The cells were subjected to 1% OGD conditions for different durations (12, 24, 36, and 48 h) for the selection of the optimal duration and with 5, 10, 20, or 50 µM API or without API for the selection of the optimal API concentration. Cell viability was evaluated with Cell Counting Kit-8 (#CK04; Dojindo, Japan), and all experiments were performed in accordance with the manufacturer’s instructions. Absorbance was measured at 450 nm with a microplate reader (Synergy H1 Hybrid Reader, BioTek, USA). The optical density values are shown as the percentages of the control values.

### Lactate dehydrogenase release assay

The release of cytoplasmic lactate dehydrogenase (LDH) is indicative of the loss of cell membrane integrity and thus represents cell death [30]. A commercial LDH cytotoxicity assay kit purchased from Beyotime, China, was used. In accordance with the manufacturer’s instructions, the LDH release agent was first added to the control sample with the maximum enzyme activity prepared in advance and allowed to be wholly lysed. Then, a 96-well plate was added with the prepared LDH working solution and incubated at 37 °C for 30 min. Lastly, the plate was measured spectrophotometrically at 600 nm with a microplate reader (Synergy H1 Hybrid Reader, BioTek, USA). The values shown are the percentages of total LDH (intracellular plus supernatant LDH).

### Western blot analysis

Retinal tissues were lysed in radioimmunoprecipitation assay buffer (ASPEN, China) containing a protease inhibitor cocktail (ROCHE). All samples were subjected to protein content determination by using a BCA assay kit (ROCHE), electrophoresed by using SDS-PAGE (ASPEN, China), and transferred to a PVDF membrane. Subsequently, the membrane was blocked with 5% skimmed milk at room temperature for 1 h, incubated with the primary antibody diluent at 4 °C overnight, then incubated with the horseradish peroxidase-conjugated secondary antibody for 1 h at room temperature. The blot was developed by using enhanced chemiluminescence and photographed in a dark room. Immune response bands were analyzed by utilizing Image J, and GAPDH and β-actin were used as the loading controls. The primary antibodies were as follows: Bax (1:1000, Abcam: ab182773), Bcl-2 (1:1000, Proteintech: 60178-1-Ig), cleaved caspase-3 (1:1000, CST: 9661), Bcl-xL (1:1000, CST: 2764); OPA1 (1:1000, CST: 80,471); DRP1 (1:1000, CST: 8570); MFN2 (1:1000, CST: 9482); GAPDH (1:1000, CST: 5174); and β-actin (1:1000, Proteintech: HRP-66,009).

### Membrane potential and mitochondrial content measurements

The ratio of aggregates/monomers of mitochondrial membrane potential (MMP) in R28 cells was assessed by using the dye JC-1 (C2003S, Beyotime, China) and the mitochondrial function was assessed by TMRE (C2001S, Beyotime, China) in accordance with the manufacturer’s instructions. Mitochondrial morphology was investigated independently by using Mito-Tracker Green (C1048, Beyotime, China). Nuclei were stained with Hoechst 33,342 (C1027, Beyotime, China). R28 cells were incubated in accordance with the manufacturer’s instructions then measured by utilizing a fluorescence microscope (Leica DMI 3000 B, Germany). Images were analyzed and merged by using ImageJ software.

### Detection of intracellular ROS levels

ROS accumulation was quantified through fluorescence detection by using the fluorescent probe 2ʹ,7ʹ-dichlorofluorescein diacetate (DCFH-DA, Beyotime, China). R28 cells were subjected to the appropriate treatments in six-well plates for 24 h then incubated for 20 min in the dark at 37 °C with 10 µM DCFH-DA solution. After incubation, the cells were imaged within 30 min by using a fluorescence microscope (Leica DMI 3000B, Germany). The fluorescence intensity (FI) of ROS was measured with a microplate reader (Synergy H1 Hybrid Reader, BioTek, USA).

### Statistical analysis

Data were presented as mean ± SD. Statistical significance was calculated with a one-way ANOVA test corrected for multiple comparisons (Tukey’s) for the comparison of samples between groups. T-test analysis was performed by using unpaired and paired Student t-tests between two groups in accordance with different methodologies. Statistical analysis was conducted with GraphPad Prism (v9.0.0, GraphPad, San Diego, CA, USA). *P* < 0.05 was considered statistically significant and marked in figures.

## Results

### API mitigated retinal damage

**Fig. 2:**
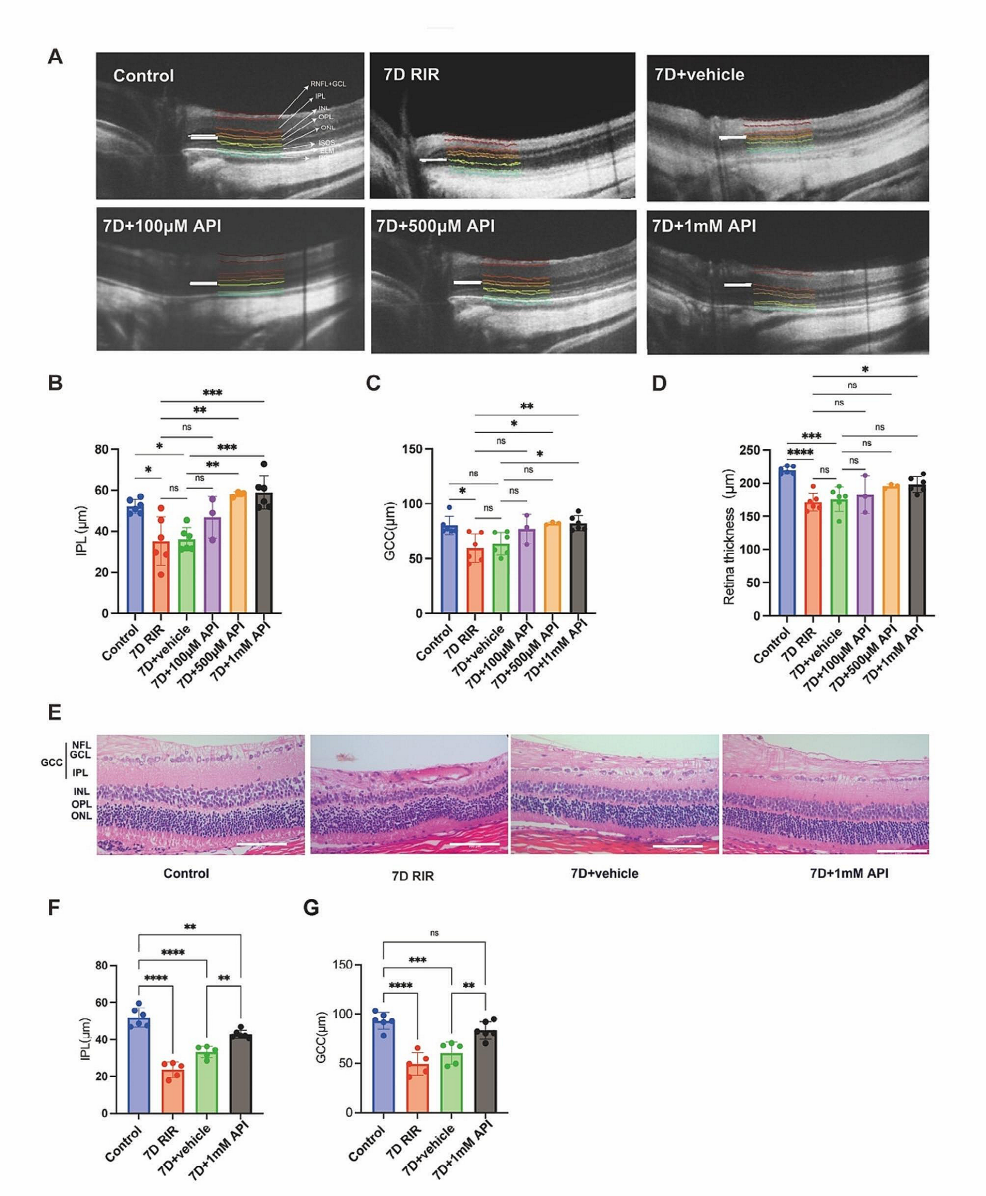
API treatment mitigated the structural impairments of the retina. **(A)** OCT images of different groups. **(B–D)** IPL, GCC, and full retinal layer thicknesses in the API-treated retina had increased relative to those in the RIR retina, and API exhibited the most pronounced effect at the concentration of 1 mM (7D + vehicle vs. 7D + API 1mM). *N* = 6 in each group. **(E)** H&E staining images of different groups. **(F–G)** API treatment increased the thicknesses of the IPL and GCC, scale bar: 100 μm. Data are shown as mean ± SD. *N* = 6 in each group. Ns, not significant. *P < 0.05, ** *P* < 0.01, ***P < 0.001, ****P < 0.0001. API, apigenin; OCT, optical coherence tomography; IPL, inner plexiform layer; GCC, ganglion cell complex; GCL, ganglion cell layer; RGC, retinal ganglion cells; NFL, nerve fiber layer; INL, inner nuclear layer; OPL, outer plexiform layer; ONL, outer nuclear layer. ELM, external limiting membrane; ISOS, inner and outer segments of the photoreceptors; RPE, retinal pigment epithelium. H&E, hematoxylin and eosin; SD, standard deviation. Note: 7D indicated that rats in each group, after undergoing respective treatments, were fed for 7 days

To investigate the optimal concentration of API, the rats were divided into control, 7D, 7D + vehicle, and 7D + API (100 µM, 500 µM, and 1 mM) groups. The 7D + vehicle group served as the negative control group corresponding to the API treatment group. There was no significant difference between the 7D group and the 7D + vehicle group on every layer of the retina (Figure S1). The results indicated that 1 mM API exhibited the most substantial protective effect, contributing to increased thickness in the IPL (*P* < 0.001) and GCC (*P* < 0.05) compared to the 7D + vehicle group **(**Fig. 2. **A-D)**. Therefore, we chose 1 mM API for subsequent experiments. H&E staining revealed that the IPL thickness was 51.92 ± 5.07 μm in the control group, 33.25 ± 3.19 μm in the 7D + vehicle group, and 42.76 ± 2.23 μm in the 7D + API group. It was observed that the IPL thickness decreased in the 7D + vehicle group; however, the 7D + API group demonstrated significant protection of the IPL (*P* = 0.0034) compared to the 7D + vehicle group. Nevertheless, it did not completely rescue the IPL layer (*P* = 0.003) (Fig. 2E). The GCL thickness was 93.22 ± 8.51 μm in the control group, 60.58 ± 11.27 μm in the 7D + vehicle group, and 83.54 ± 8.88 μm in the 7D + API group. It was observed that the thickness of the GCC layers decreased in the 7D + vehicle group, while the 7D + API group significantly protected the GCC layers (*P* = 0.0065) (Fig. 2G).

### API increased RGCs survival

**Fig. 3:**
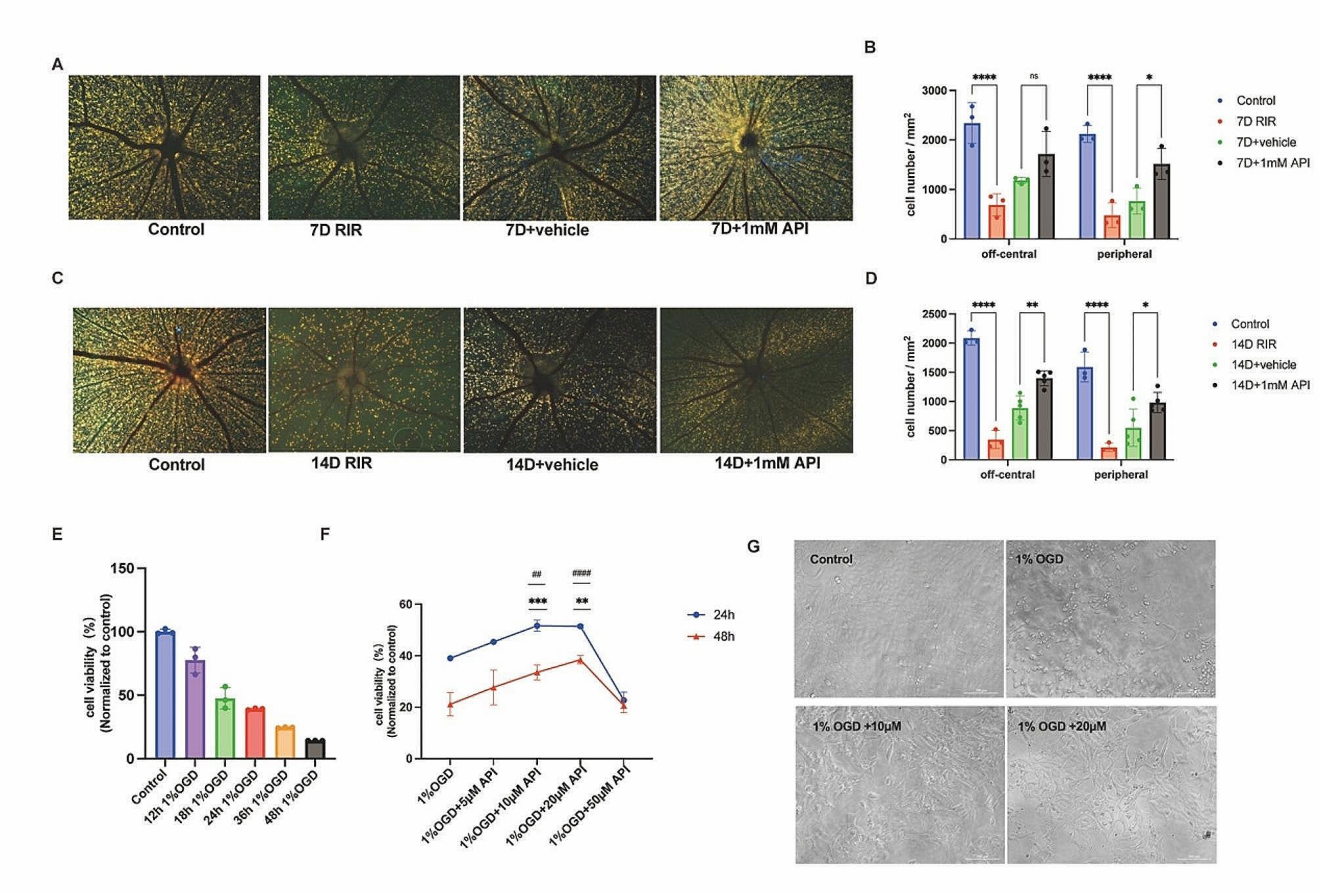
API increased RGC survival and cell viability. **(A)** Representative fluorescence images of labeled surviving RGCs at one week. **(B)** API treatment mitigated significant RGC loss in peripheral areas at one week (*P* < 0.05). *N* = 3 in each group. **(C)** Representative fluorescence images of labeled surviving RGCs at day at two weeks. **(D)** API treatment mitigated significant RGC loss in off-central (*P* < 0.01) and peripheral areas (*P* < 0.05) at two weeks. *N* = 3 in each group. **(E)** R28 cells were subjected to 1% OGD conditions for different durations. *N* = 3 in each group. **(F)** R28 cells were treated with API (0–50 µM) for 24 h and 48 h, and 10 µM and 20µM API showed the most pronounced effect. *N* = 3 in each group. Ns, not significant, *** 1% OGD vs. 1% OGD + 10µM at 24 h. ^##^ 1% OGD vs. 1% OGD + 10 M at 48 h. ** 1% OGD vs. 1% OGD + 20µM at 24 h. ^####^ 1% OGD vs. 1% OGD + 20µM at 48 h. (G) Cell morphology was shown with a biological microscope at 24 h. Data are shown as mean ± SD. *P < 0.05, **P < 0.01, ***P < 0.001, ****P < 0.0001. RGC, retinal ganglion cell; OGD, oxygen-glucose deprivation. Note: 14D indicated that rats in each group, after undergoing respective treatments, were fed for 14 days

To investigate the long-term effect of API on the survival of RGCs, FG was employed to label RGCs with intact axons on day 7 and day 14. On day 7, the mean densities of RGCs in the off-central and peripheral regions of the retina were 2,341.67 ± 409.55 cells/mm2 and 2,123.92 ± 172.04 cells/mm2, respectively, in the control group. The mean densities of RGCs were 1180.38 ± 64.02 cells/mm2 and 763.44 ± 262.85 cells/mm2, respectively, in the 7D + vehicle group. The mean densities of RGCs in the 7D + API group were 1716.40 ± 454.66 cells/mm2 and 1515.05 ± 311.63 cells/mm2, respectively. These results showed that compared with the 7D + vehicle group, API had a significant protective effect on RGC survival in the peripheral region (*P* = 0.03), but not in the off-central region (*P* = 0.15) (Fig. 3A, B). On day 14, the mean densities of RGCs were 2089.25 ± 121.15 cells/mm2 and 1593.01 ± 256.86 cells/mm2, respectively, in the control group. In the 14D + vehicle group, the mean densities of RGCs were 888.06 ± 207.04 cells/mm2 and 549.09 ± 319.78 cells/mm2, respectively. The densities of RGCs in the central and peripheral regions of the retina in the 14D + API group were 1165.21 ± 582.10 cells/mm2 and 817.93 ± 429.48 cells/mm2, respectively. API was found to have a protective effect on both the off-central (*P* = 0.0038) and peripheral (*P* = 0.014) regions when compared with the 14D + vehicle group (Fig. 3C, D).

Furthermore, R28 cells were subjected to 1% OGD conditions for different durations and obtained 50% viability of normal cells at 24 h (Fig. 3E). API treatment showed a dose-dependent increase in viability of 1% OGD-induced R28 cells, with a peak effect observed at 10 µM (*P* < 0.001) and 20 µM (*P* < 0.001) at 24 h and 48 h, respectively, while concentrations exceeding 50 µM appeared to result in toxicity at 48 h (Fig. 3F). Cell morphology was also observed using a biological microscope at 24 h and displayed more intact cellular morphology (Fig. 3G).

### API enhanced RGCs function and was safe for normal RGCs

**Fig. 4:**
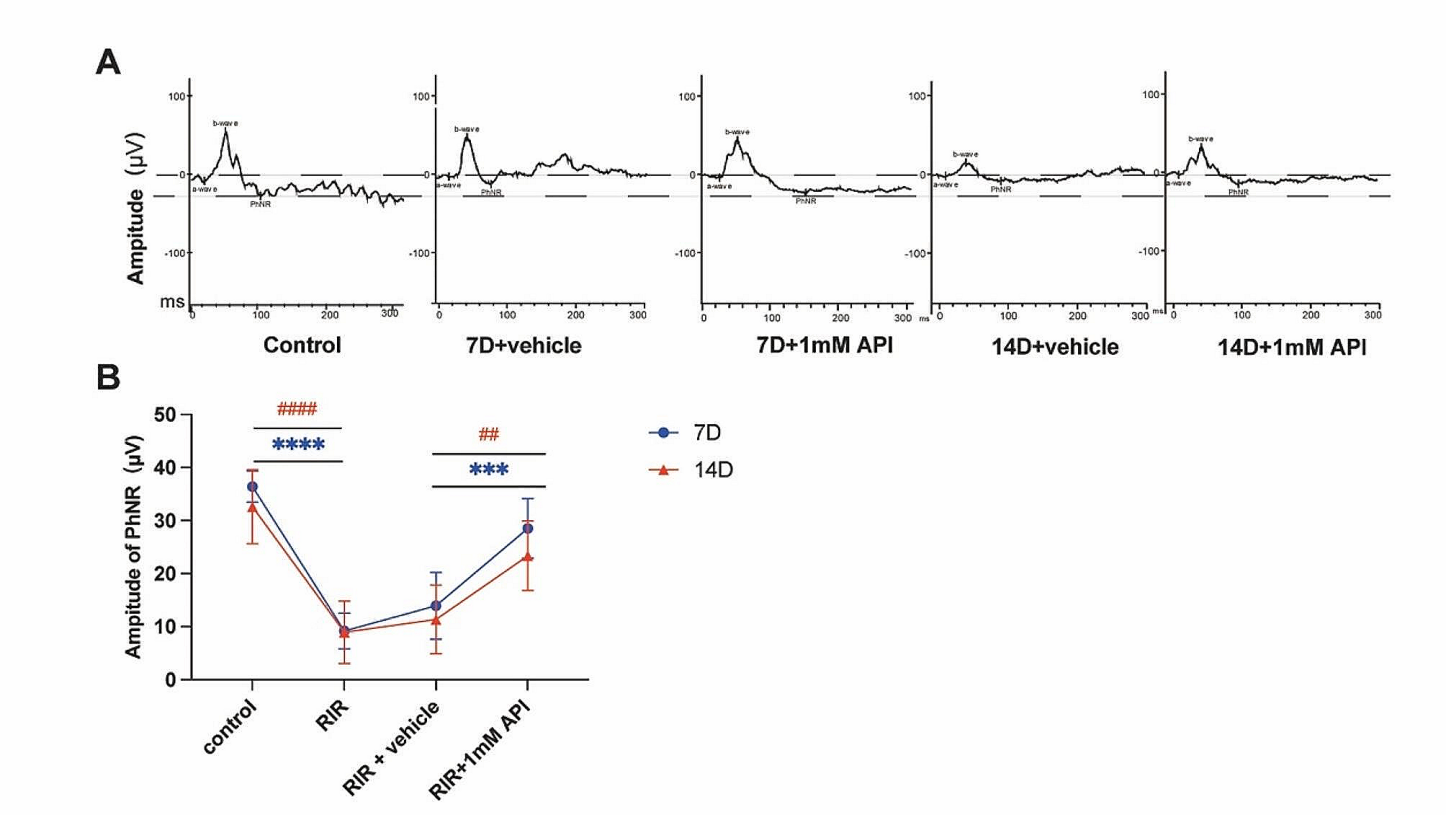
API increased RGC function. **(A)** Representative PhNR images of different groups at one week and two weeks, respectively. **(B)** Quantitative analysis of PhNR amplitudes at days 7 and 14. RIR injury dramatically reduced the amplitudes of PhNR. This effect was alleviated by API treatment. *N* = 6 in each group. Data are shown as mean ± SD. **P < 0.01, ^##^*P* < 0.01; ***P < 0.001, ****P < 0.0001. RIR, retinal ischemia-reperfusion. PhNR, photopic negative response

**Fig. 5:**
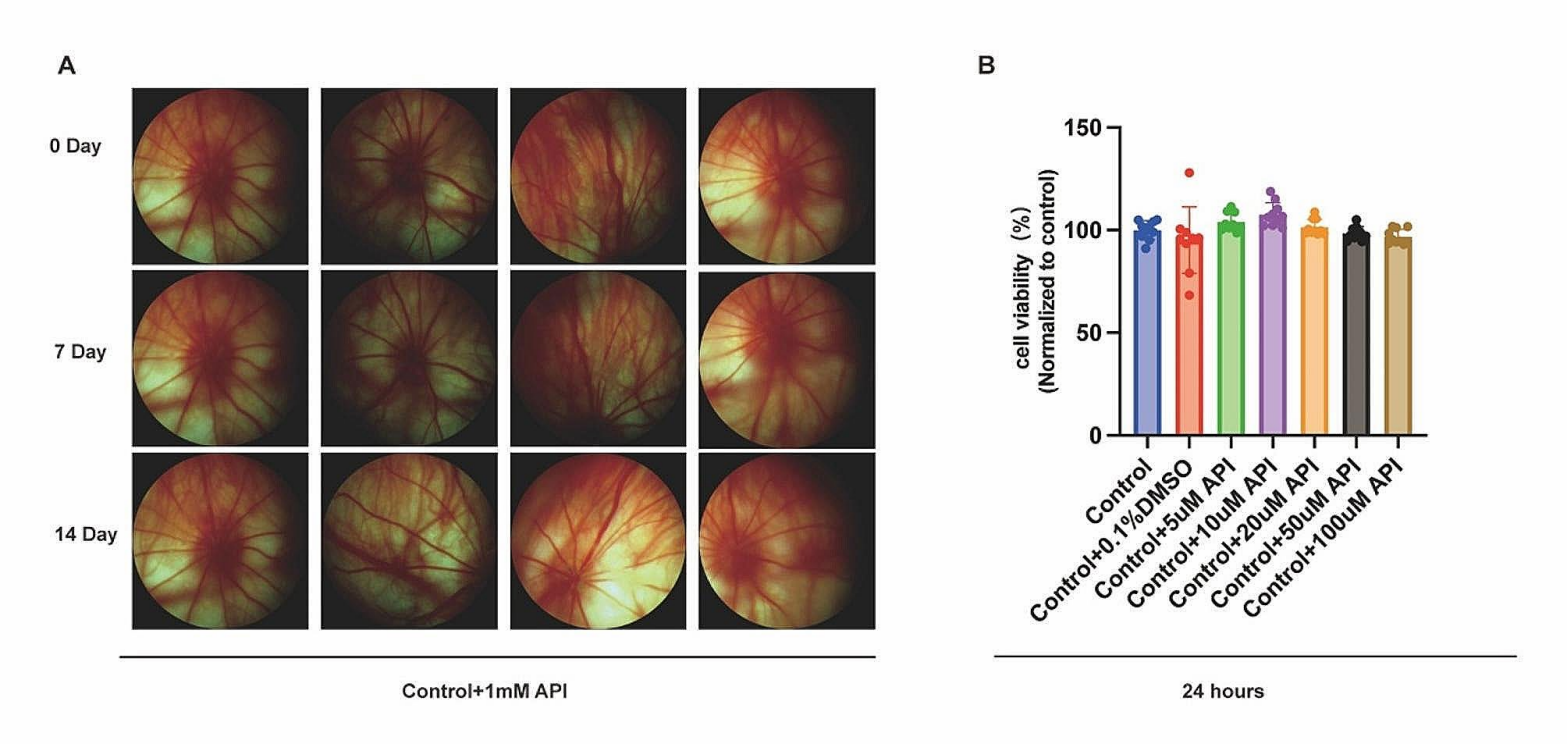
API was safe for intravitreous injection and co-incubation. **(A)** Fundus photos of the same rat showed that API can be safely intravitreously injected over time. *N* = 4 in the Control + 1 mM API group. **(B)** API showed no cytotoxicity toward normal R28 cells. The control group represented normal cells without any treatment. The other six groups underwent respective treatments in normal cells. All groups were cultured together for 24 h. *N* = 9 in each group. Note: 0 Day indicated that rats in the Control + 1mM API group, after intravitreal injection of 1 mM API, underwent fundus imaging on the same day

Next, we examined PhNR, a well-established measure of retinal ganglion cell (RGC) function [31]. As shown in Fig. 4A, B. API treatment significantly attenuated the decrement in PhNR amplitude at day 7 (*P* = 0.0004) and 14 (*P* = 0.0064), supporting the notion that API has the potential to preserve RGC function in a long period. Importantly, we further verified the safety of API. Continuous retinal imaging revealed no significant adverse events in the fundus of normal rats (Fig. 5A), indicating that API is well-tolerated in vivo. In addition, we investigated the toxicity of API towards normal R28 cells by assessing cell viability. We found that different concentrations of API (5-100 µM) had no significant toxic effects on the cell viability of normal cells (Fig. 5B).

### API inhibited the apoptosis of RGC

**Fig. 6:**
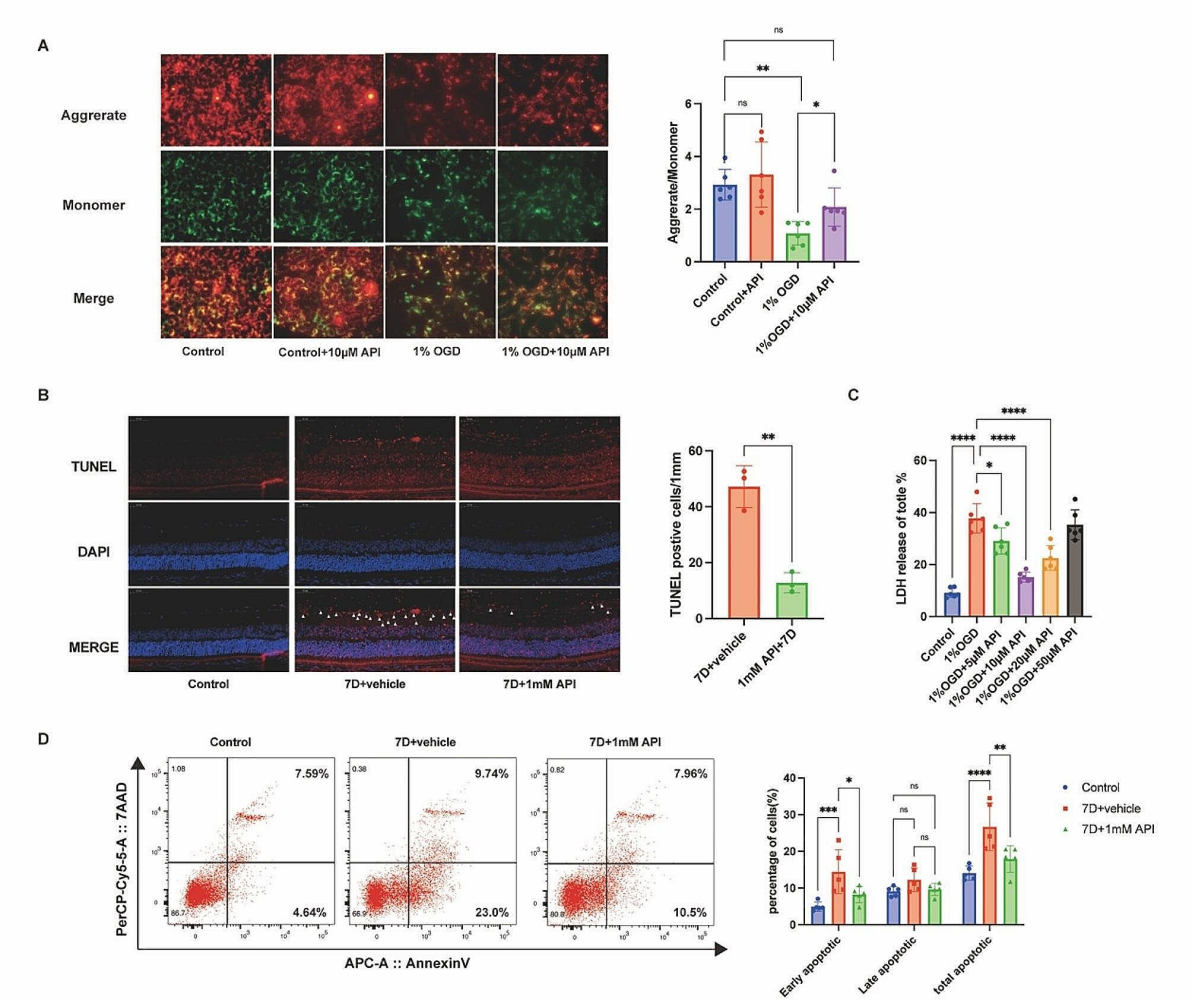
API inhibited RGC apoptosis. **(A)** API increased the aggregate/monomer ratio. Green fluorescence: JC-1 monomers; Red fluorescence: JC-1 aggregates. Scale bar: 20 μm. *N* = 6 in each group. **(B)** API treatment decreased the number of TUNEL-positive cells in the GCL. The white triangle points to TUNEL-positive cells. *N* = 3 in each group. **(C)** R28 cells were treated with API (0–50 µM) for 24 h, and 10 μm API showed the most pronounced effect. *N* = 6 in each group. **(D)** API decreased the percentage of apoptotic cells, compared with the 7D + vehicle treatment, especially the early apoptotic cells. *N* = 5 in each group. Data are shown as mean ± SD. Ns, not significant, *P < 0.05, **P < 0.01. TUNEL, TdT-mediated dUTP nick-end labeling; LDH, lactate dehydrogenase

Early apoptosis was detected first. The ratio of aggregates/monomers decreased significantly (Control vs. 1% OGD, *P* < 0.01) after incubating with API for 24 h, as shown in Fig. 6A. API increased the early cell viability (1% OGD vs. 1% OGD + 10 μm API, *P* < 0.05), indicating API decreased early apoptosis cells in 1% OGD induced R28 cells. Following a seven-day treatment, API was found to reduce cell apoptosis compared to the 7D + vehicle group, as indicated by TUNEL staining (*P* = 0.002) (Fig. 6B). The expression levels of apoptosis-related proteins were analyzed over time after RIR. Furthermore, supernatants from the R28 cells were analyzed for cell death, as measured by LDH release. Treatment with API at concentrations ranging from 5 to 20 µM significantly decreased the release of LDH, with 10 µM API demonstrating the most significant effect (*P* < 0.0001), indicating a potential protective effect of API on cells (Fig. 6C). The apoptosis rate was also determined by flow cytometry assay, where API was observed to decrease the percentage of apoptotic cells, particularly early apoptotic cells, compared to the 7D + vehicle treatment (*P* = 0.025) (Fig. 6D).

**Fig. 7:**
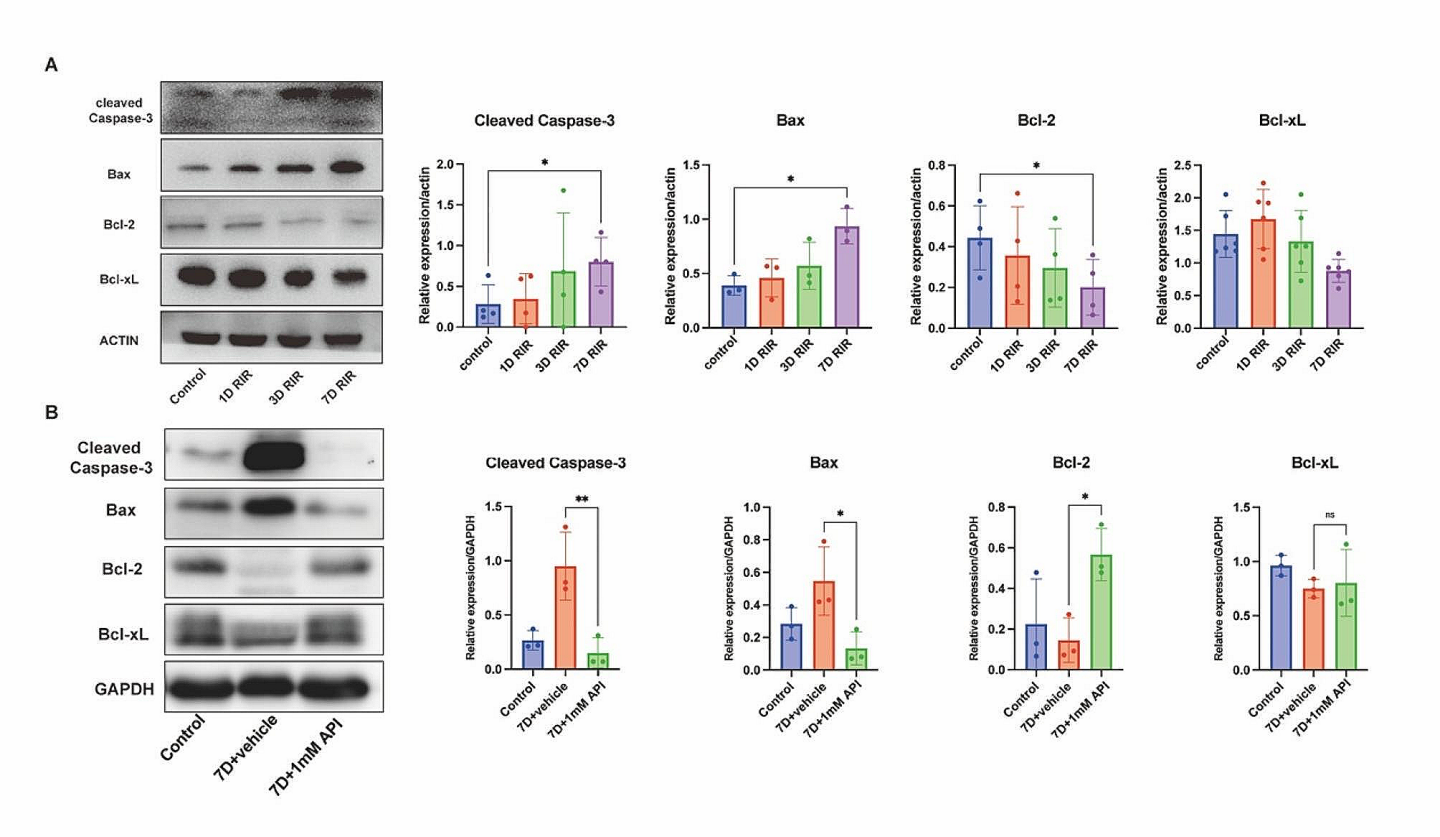
API regulated the expression of apoptosis-related proteins. **(A)** Cleaved caspase-3 and Bax expression increased, whereas Bcl-2 and Bcl-xL expression decreased over time. *N* = 3 in each group. **(B)** API inhibited RGC apoptosis as reflected by Bcl-2, Bcl-xl, Bax, and cleaved caspase-3 expression. *N* = 3 in each group. Data are shown as mean ± SD. *P < 0.05, **P < 0.01. Note: 1D and 3D indicated that rats in each group, after undergoing respective treatments, were fed for 1 day and 3 days, respectively

As illustrated in Fig. 7A, the expression levels of cleaved caspase-3 (*P* = 0.017) and Bax (*P* = 0.016) increased over time, while those of anti-apoptotic proteins Bcl-xL (*P* = 0.209) and Bcl-2 (*P* = 0.037) decreased over time. Therefore, a seven-day duration was chosen for further analysis of the effect of API on apoptosis. Figure 7B showed that the expression of cleaved caspase-3 was significantly upregulated (*P* = 0.015) in the 7D + vehicle group compared to that in the control group, and was 6.3-fold higher than that in the 7D + API group (*P* = 0.0072). Additionally, the expression levels of Bax and Bcl-2 in the 7D + vehicle group were 4.23 times (*P* = 0.031) and 0.26 times (*P* = 0.04) higher than those in the 7D + API group, respectively. While Bcl-xL was downregulated, no notable differences were observed between the 7D + vehicle and 7D + API groups (*P* = 0.93).

### API restored mitochondrial crista morphology and mitochondrial integrity

**Fig. 8:**
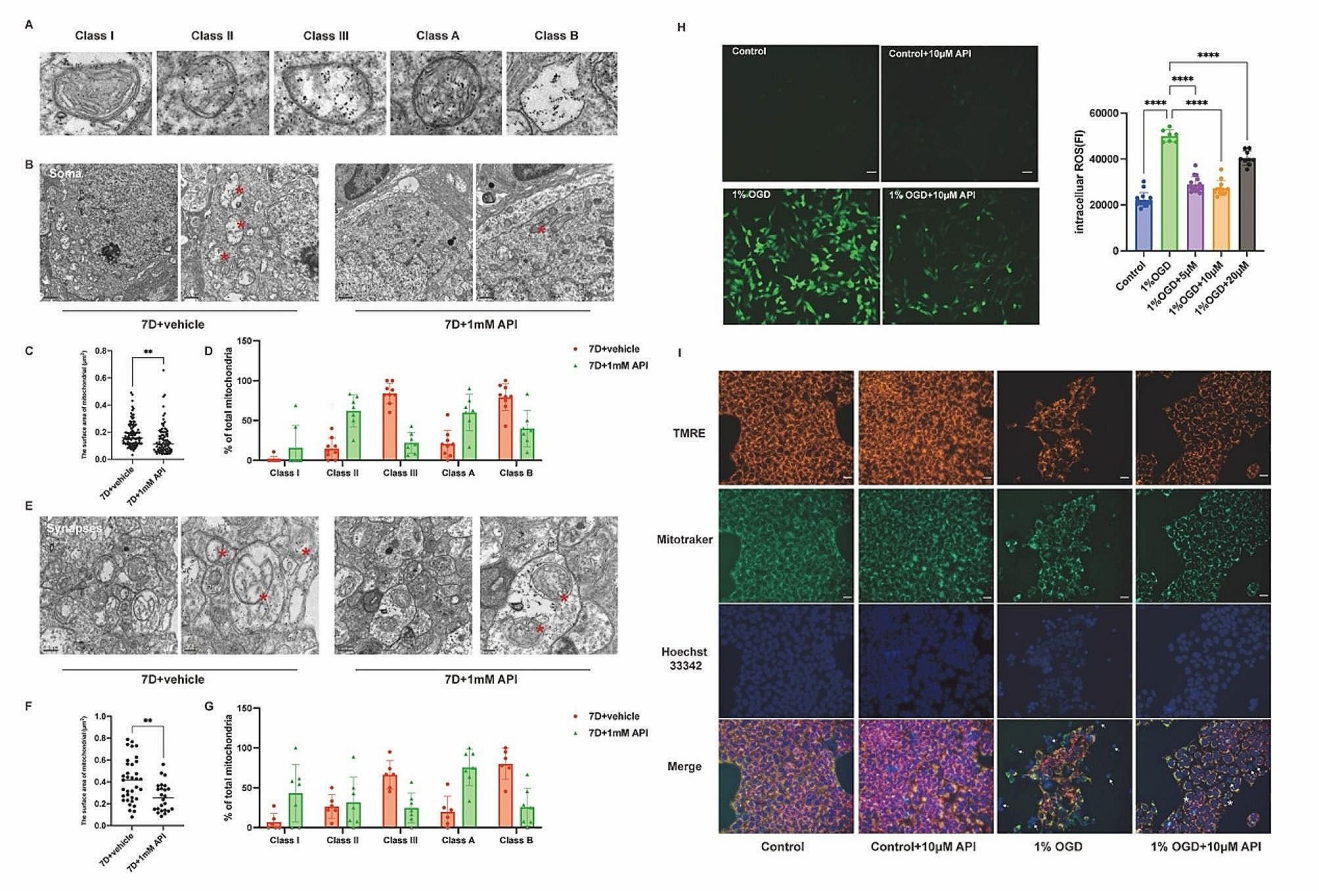
API restored mitochondrial crista morphology and mitochondrial integrity and increased mitochondrial function. **(A)** Mitochondria in the GCC were grouped in accordance with mitochondrial crista number, matrix density, and swelling degree. **(B, E)** Representative TEM images of the mitochondria of RGC soma and synapses in different experimental groups. Red asterisks point to mitochondria. **(C, F)** Surface area of the mitochondria of RGC soma and synapses in different experimental groups. *N* = 3 in each group. **(D, G)** A total of 10–30 mitochondria per experiment were divided into three categories in accordance with crista number: Class I (more than four cristae), Class II (two or three cristae), and Class III (no more than one cristae); 10–30 mitochondria per experiment were divided into two categories in accordance with matrix density and swelling degree: Class A (mitochondria with a dense matrix) and Class B (mitochondria with a hypodense matrix). Scale bar: 1 μm-500 nm. *N* = 3 in each group. **(H)** API decreased the release of mitochondrial ROS. Scale bar: 40 μm. *N* = 6 ∼ 9in each group. **(I)** API increased mitochondrial function. White arrows point to cells with the almost complete loss of mitochondria and complete loss of MMP. Asterisks indicate cells that retained their mitochondria but lost their MMP. *N* = 3 in each group. Scale bar: 20 μm. Data are shown as mean ± SD. *P < 0.05, **P < 0.01. ROS, reactive oxygen species; MMP, mitochondrial membrane potential

We utilized transmission electron microscopy (TEM) to evaluate the therapeutic effects of API on mitochondrial ultrastructure following renal ischemia-reperfusion (RIR) injury. Specifically, we investigated changes in mitochondrial ultrastructure seven days after RIR and separately observed the soma and synapses of retinal ganglion cells (RGCs) in the ganglion cell complex (GCC) layer. Our results, as depicted in Fig. 8C **and F**, showed that treatment with API resulted in a smaller mitochondrial surface area of the soma and synapses compared to the 7D + vehicle group (*P* < 0.01). Mitochondria were classified into different categories based on previous studies [32], including Class I (more than four cristae), Class II (two or three cristae), and Class III (no more than one cristae) and Class A (mitochondria with a dense matrix) and Class B (mitochondria with a hypodense matrix) **(**Fig. 8A). In the 7D + vehicle group, extensive mitochondrial damage was observed in the soma (Fig. 8B), with approximately 80.1% and 79.7% of the mitochondria classified as Classes III and B, respectively. However, treatment with API significantly reduced the ultrastructural damage of abnormal mitochondria, resulting in approximately 62.2% Class II and 60.1% Class A mitochondria (Fig. 8D). Similar results were observed in synapses, where treatment with API resulted in approximately 24.8% Class III and 25.8% Class B mitochondria, as opposed to approximately 66.3% Class III and 80% Class B mitochondria in the vehicle group (Fig. 8G). These findings suggested that API was effective in restoring mitochondrial ultrastructure after RIR injury. Mitochondrial-derived ROS are a crucial source of cytosolic ROS, prompting us to investigate ROS production. Our results showed a significant increase in ROS production under 1% OGD conditions compared to control conditions. However, pretreatment with 5–20 µM API substantially prevented ROS accumulation (Fig. 8H). Given that API significantly reduced ROS generation, we speculated that it could protect R28 cells by improving mitochondrial function and structure. To further investigate this hypothesis, we observed mitochondrial function and found that API treatment resulted in reduced mitochondrial loss under 1% OGD conditions (Fig. 8I).

### API modulated mitochondrial dynamics

**Fig. 9:**
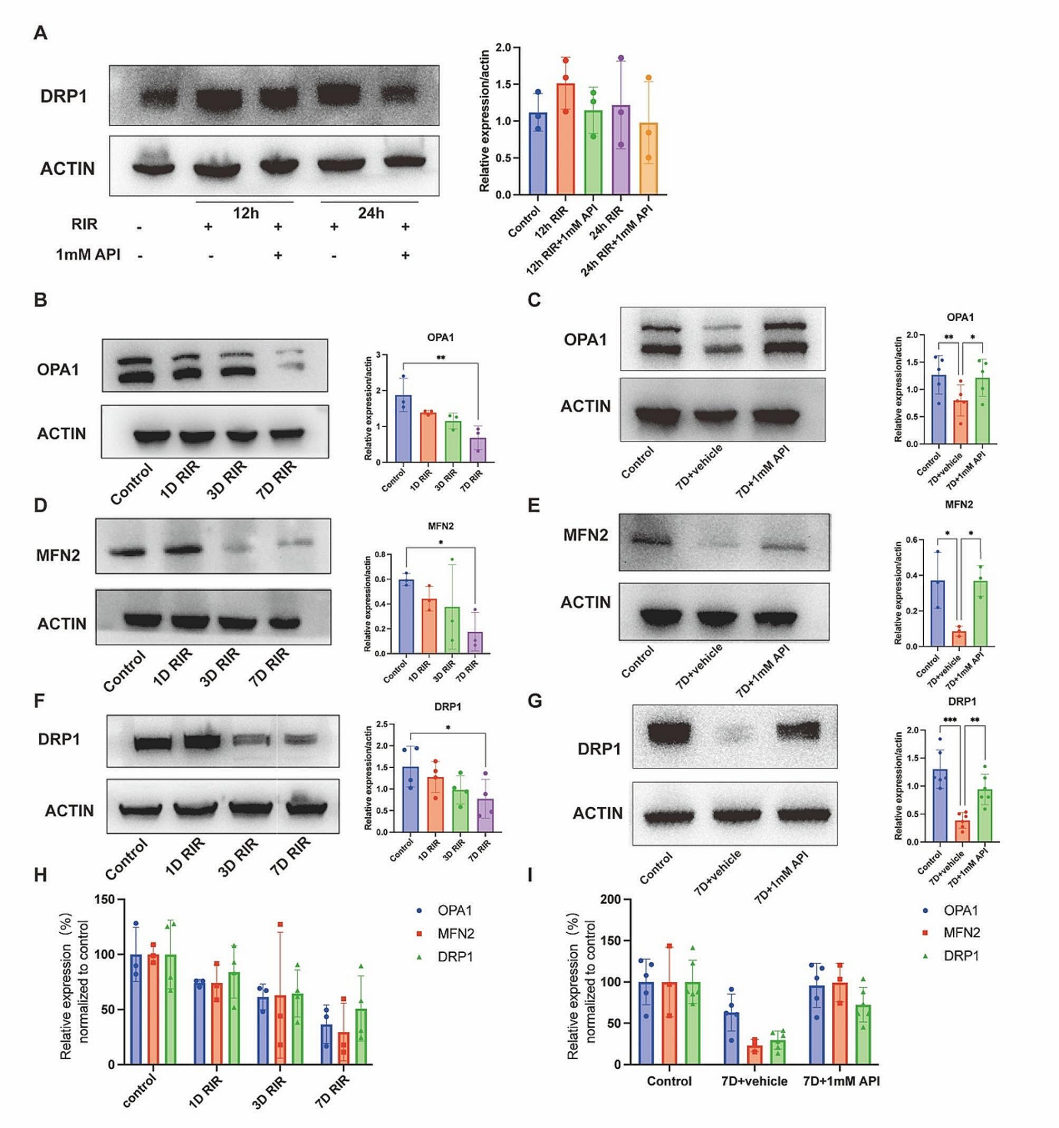
API modulated the expression of proteins involved in mitochondrial dynamics. **(A)** Western blot analysis showed that API downregulated DRP1 expression at 12 and 24 h after RIR. **(B)** DRP1 expression gradually decreased from day 1 to day 7. **(C)** Western blot analysis demonstrated that API upregulated DRP1 expression at day 7 after RIR. **(D, F)** Expression of the mitochondrial fusion proteins OPA1 and MFN2 decreased from day 1 to day 7. **(E, G)** Western blot analysis showed that API upregulated OPA1 and MFN2 expression at day 7 after RIR. **(H-I)** Expression levels of OPA1, MFN2, and DRP1 were compared with normal values at different time points and in different groups. *N* = 3 in each group. Data are shown as mean ± SD. *P < 0.05, **P < 0.01, ***P < 0.001, ****P < 0.0001. OPA1, optic atrophy 1; MFN2, mitofusin-2; DRP1, dynamin-related protein-1

**Fig. 10:**
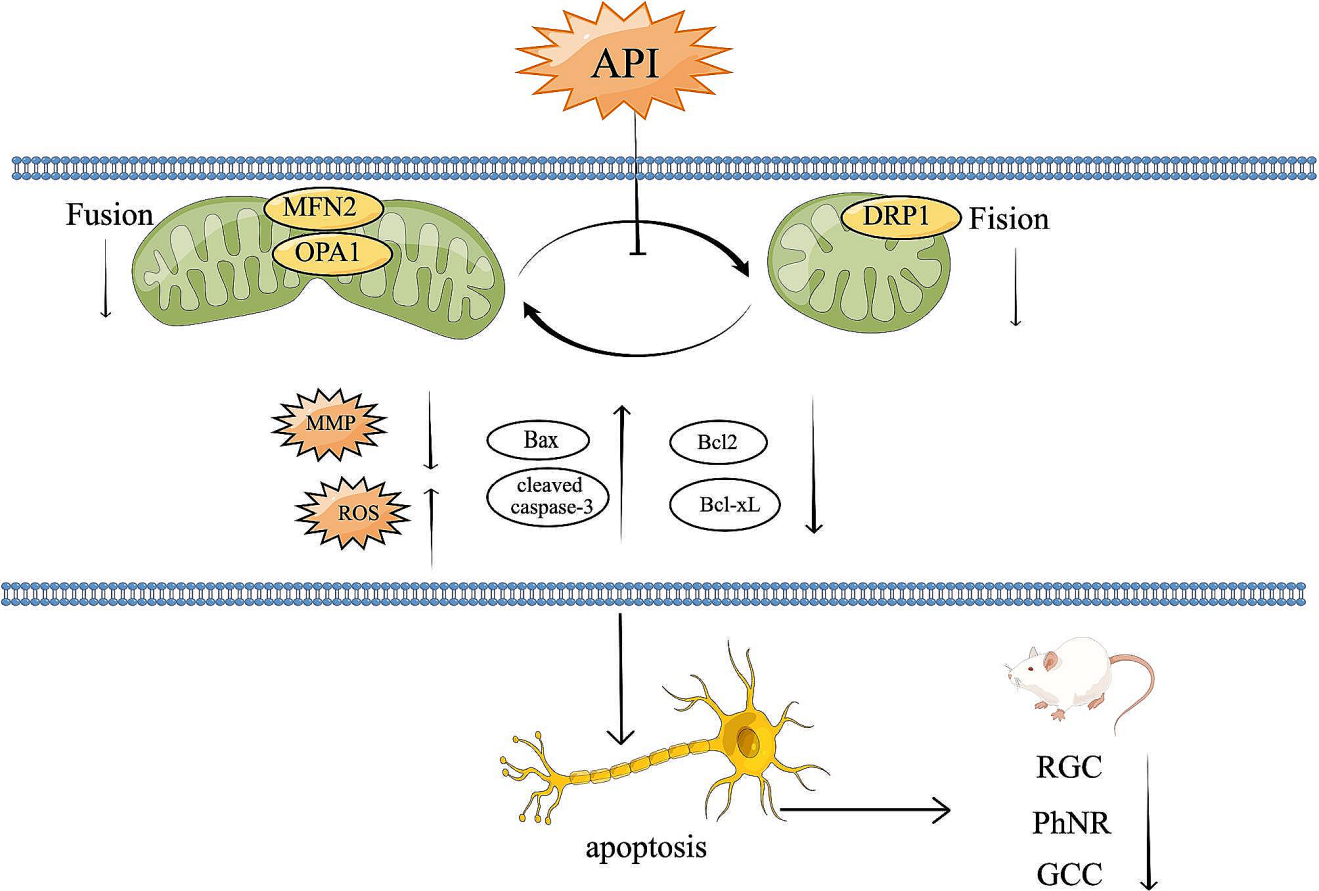
Schematic summarizing the effects of API administration on mitochondrial dynamics, apoptosis, RGC survival, and RGC function in RIR injury

Mitochondrial dysfunction caused by RIR injury may result from an imbalance in mitochondrial dynamics. Therefore, we investigated the expression of proteins associated with mitochondrial dynamics. DRP1 expression was examined at 12 and 24 h after RIR injury and found that API treatment reduced DRP1 expression, although the effect was not significant (*P* > 0.05) (Fig. 9A). Additionally, we evaluated DRP1 expression from days 1 to 7 and found that it decreased over time (*P* = 0.0276) (Fig. 9B). API treatment increased DRP1 expression on day 7 (*P* = 0.0013) (Fig. 9C). The expression levels of the mitochondrial fusion proteins OPA1 and MFN2 decreased over time after RIR injury and were significantly reduced on day 7 (Fig. 9D, F). API treatment increased the expression levels of OPA1 (*P* = 0.040) and MFN2 (*P* = 0.0375) (Fig. 9E, G). Furthermore, we compared the degree of reduction in mitochondrial fusion and fission based on protein expression and found that the expression of mitochondrial fission proteins decreased to a lesser extent than that of mitochondrial fusion proteins (Fig. 9H). API treatment normalized the expression of mitochondrial fusion proteins and partially increased the expression of mitochondrial fission proteins (Fig. 9I). In conclusion, Fig. 10 showed the effects of API administration on mitochondrial dynamics, apoptosis, RGC survival, and RGC function in RIR injury.

## Discussion

The results of this study demonstrated that API reduced RIR-induced inner retina histological damage and improved RGC survival and function while reducing apoptosis by modulating mitochondrial dynamics. These findings revealed the protective effect of API against RGC degeneration caused by RIR.

API effectively protects various organs, such as the brain, kidney, myocardium, and liver, from ischemia/reperfusion injury [33–36]. For example, Huang et al. found that in myocardial ischemia/reperfusion injury, API improved cardiac function by decreasing apoptosis in vivo and in vitro [34]. Pang et al. suggested that in MCAO/R rats, API may protect the brain against ischemia/reperfusion injury by attenuating brain damage and improving neurological function [35]. Similarly, in this study, API effectively protected against RIR injury. Furthermore, studies have reported that API exerted a protective effect on ARPE-19, BV-2, and SH-SY5Y cells under an OGD condition in vitro [37–39]. Consistent with the above findings, our results demonstrated that API positively affected 1% OGD-induced R28 cells. Furthermore, previous studies have shown that API enhanced the retinal blood barrier, protected against bright light-induced photoreceptor degeneration, and protected the oxidative-induced mouse retina [19, 20, 40]. We further discovered that API mitigated RIR-induced tissue damage, improved RGC survival, and ameliorated retinal dysfunction. Overall, API appears to be a promising RGC protective agent.

Normal neuronal function may depend on the good balance between mitochondrial fission and fusion [41]. Zhang et al. reported that in RGC-5 cells, resveratrol treatment decreased apoptosis by upregulating AMPK, NRF-1, Tfam, MFN2, and OPA1 expression [42]. Consistent with our study, our work found that API upregulated OPA1 and MFN2 after RIR injury, indicating that the promotion of mitochondrial fusion may be a promising target. Shields et al. reported that DRP1 depletion caused axonal mitochondrial dysfunction, thus resulting in neuronal death [41]. However, another study reported that in DBA/2J mice, DRP1 inhibition ameliorated oxidative stress-mediated division and dysfunction in RGCs and their axons during glaucomatous neurodegeneration [43]. Hence, the current research suggests that excessive and untimely fission processes are detrimental and that normal neuronal function may depend on the fine balance of mitochondrial fission [41]. In RIR injury, mitochondrial fission increased in a short period and reduced as the injury progressed over time, and API acted to alleviate mitochondrial imbalance.

Apoptosis inhibition is a promising clinical target for preventing RGC degeneration. Anti-apoptotic Bcl-2 family protein including Bcl-2 and Bcl-xL downregulate in the RGC death [44]. Among pro-apoptotic family members, Bax, existing in the membrane of mitochondria, plays a pivotal role in the regulation of RGC [45]. In the intrinsic apoptosis pathways, impaired mitochondria released cytochrome C together with Apaf-1 and procaspase-9 to form the apoptosome thus activating downstream cleaved caspase-3, and while in the extrinsic, the intracellular adaptor protein Fas-associated death domain which in turn recruits procaspase-8, thus leading to subsequent activation of cleaved caspase-3 showing cleaved caspase-3 is the common protein of both apoptosis pathways [46]. Prior studies have shown that in many diseases, API exerts a protective effect by modulating apoptosis pathways [39, 47, 48]. Consistent with previous studies, our results indicated that API inhibited RGC apoptosis after RIR.

Our study has limitations. We initially considered intravitreal injection in this study to explore the protective effect of the API due to its poor solubility (1.35 µg/mL) [49]. However, this contradicts the recommendation of the API as an oral supplement [15, 49, 50]. Fortunately, in recent years, different delivery systems (liposomes, polymer micelles, and nanosuspensions) have helped improve the solubility and stability of poorly water-soluble drugs [15]. In particular, different API-loaded carriers have been developed to improve the solubility and bioactivity of API [20, 51, 52]. Next, to further explore the therapeutic efficacy of API, alternative routes of administration such as intraperitoneal injection or oral administration could be considered in our study.

## Conclusions

The findings of this study indicated that API treatment may have a protective effect on retinal structure and function following RIR. Furthermore, it appeared that the neuroprotective mechanism of API involved the regulation of mitochondrial dynamics by increasing the expression of DRP1, OPA1, and MFN2.

### Electronic supplementary material

Below is the link to the electronic supplementary material.

**Supplementary Material 1**: **Figure S1**. Comparison of the retinal thickness of each layer in different groups. IPL, inner plexiform layer; GCC, ganglion cell complex; GCL, ganglion cell layer; RGC, retinal ganglion cells; NFL, nerve fiber layer; INL, inner nuclear layer; OPL, outer plexiform layer; ONL, outer nuclear layer. ELM, external limiting membrane; ISOS, inner and outer segments of the photoreceptors; RPE, retinal pigment epithelium. N=6 in each group. Data are shown as mean ± SD. Ns, not significant, *P < 0.05, ** *P* < 0.01, ***P < 0.001.

**Supplementary Material 2**: Explanation regarding animal experiments

## Data Availability

The original contributions presented in the study are included in the article/Supplementary Material, further inquiries can be directed to the corresponding authors.
