# Supplementary figures and images for: Neuroprotective effects of apigenin on retinal ganglion cells in ischemia/reperfusion: modulating mitochondrial dynamics in in vivo and in vitro models
Source: J Transl Med. 2024 May 13;22:447. doi: 10.1186/s12967-024-05260-1 (PMC11089678; doi:10.1186/s12967-024-05260-1)

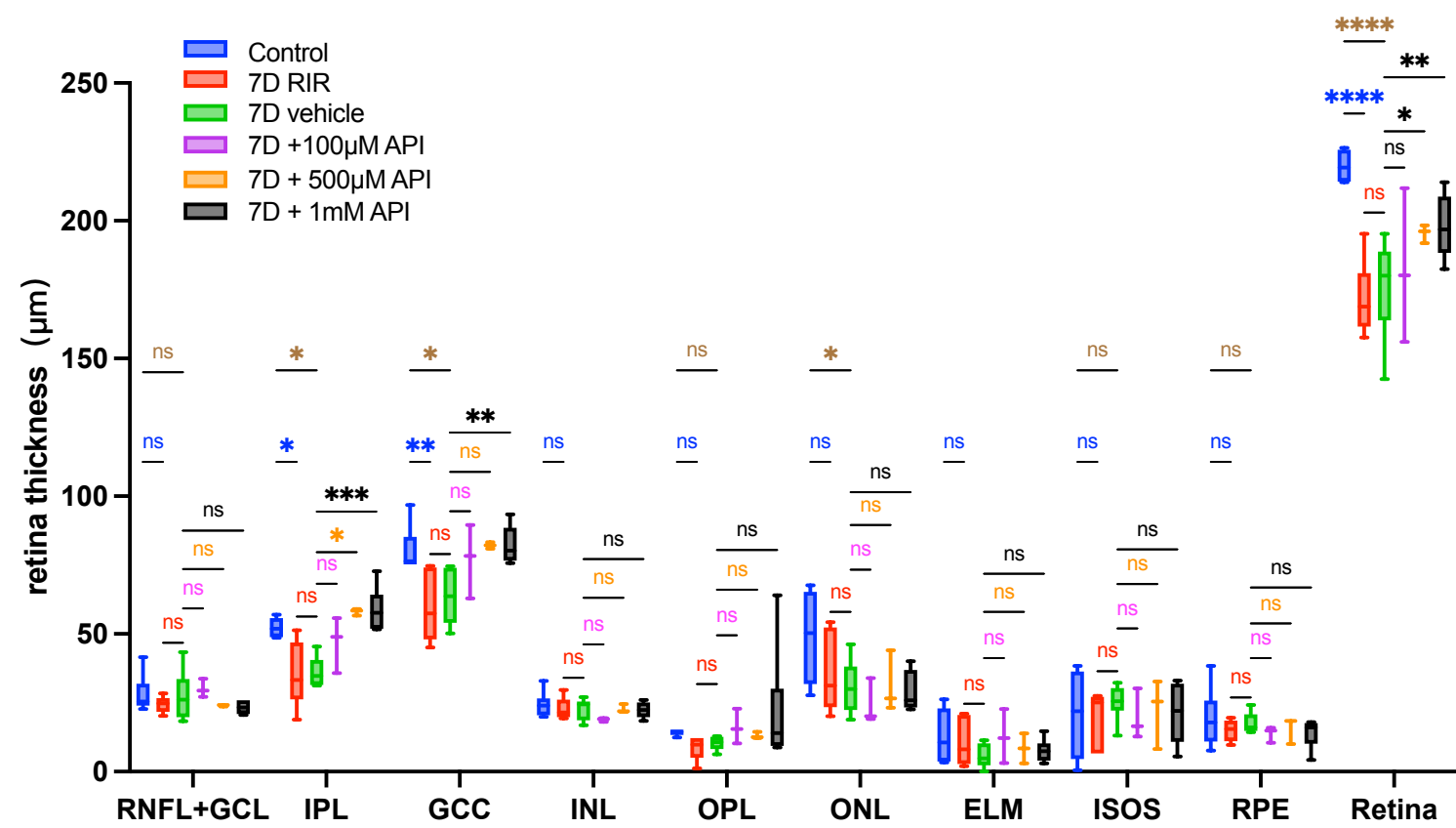

Supplement: Supplementary file 1 — Supplementary Material 1: Figure S1. Comparison of the retinal thickness of each layer in different groups. IPL, inner plexiform layer; GCC, ganglion cell complex; GCL, ganglion cell layer; RGC, retinal ganglion cells; NFL, nerve fiber layer; INL, inner nuclear layer; OPL, outer plexiform layer; ONL, outer nuclear layer. ELM, external limiting membrane; ISOS, inner and outer segments of the photoreceptors; RPE, retinal pigment epithelium. N=6 in each group. Data are shown as mean ± SD. Ns, not significant, *P < 0.05, ** P < 0.01, ***P < 0.001. [file 12967_2024_5260_MOESM1_ESM.pdf]
