# Supplementary material for: Neuroprotective effects of apigenin on retinal ganglion cells in ischemia/reperfusion: modulating mitochondrial dynamics in in vivo and in vitro models
Source: J Transl Med. 2024 May 13;22:447. doi: 10.1186/s12967-024-05260-1 (PMC11089678; doi:10.1186/s12967-024-05260-1)
Supplement: Supplementary file 2 — Supplementary Material 2: Explanation regarding animal experiments [file 12967_2024_5260_MOESM2_ESM.docx]

**Explanation regarding animal experiments**

In conducting phenotype experiments (including OCT, fundus photography, PhNR, flow cytometry), we pre-set 6 animals per group. However, in the fundus photography experiment, we began each set of fundus photography experiments with 6 rats. The experiment involved imaging each rat three times at baseline, 7 days, and 14 days. The procedure required exposing the animals to light and maintaining continuity in imaging the same retinal area. Each imaging session lasted approximately 30 minutes. Unfortunately, this prolonged exposure and repeated imaging may have stressed the animals. Two rats died during the follow-up period: one on day 7 and one on day 14. Ultimately, 4 rats were used for presentation, which did not affect the conclusions. Concerning the flow cytometry experiment, due to sample quality issues encountered during retinal sampling, data from one rat in the RIR+API group was unusable. Additionally, one rat from the RIR group died during the modeling process, possibly due to poor tolerance to anesthesia, resulting in accidental death. To maintain consistency in data analysis, one data point was randomly removed from the Control group, resulting in a sample size of n=5 for each group. Despite the reduction in sample size, our statistical analysis still showed significant differences between the groups, supporting the issues we sought to address. Given the reliability of the existing data and the challenges of replicating experiments, we have decided to continue using the data from the current experiments.

We are committed to optimizing operational procedures to shorten the duration of fundus photography for rats and closely monitoring their condition during the experiment to minimize stress and ensure their comfort and well-being. We pledge to maintain transparency and accuracy in our research and reporting.
